# The 2 faces of plant SUMOylation against viruses

**DOI:** 10.1371/journal.ppat.1012701

**Published:** 2024-12-17

**Authors:** Blanca Sabarit, Eduardo R. Bejarano

**Affiliations:** Instituto de Hortofruticultura Subtropical y Mediterránea “La Mayora” (IHSM “La Mayora”), Universidad de Málaga-Consejo Superior de Investigaciones Científicas (UMA-CSIC), Campus Teatinos, Málaga, Spain; Shanghai Center for Plant Stress Biology, CHINA

## Introduction

Like most organisms, plants are equipped with complex and sophisticated molecular mechanisms to cope with a changing environment. Among posttranslational modifications (PTMs), the conjugation of small peptides, such as ubiquitin or SUMO (small ubiquitin-related modifier), enables a rapid and efficient adaptation to a wide range of abiotic and biotic stress conditions.

**Fig 1:**
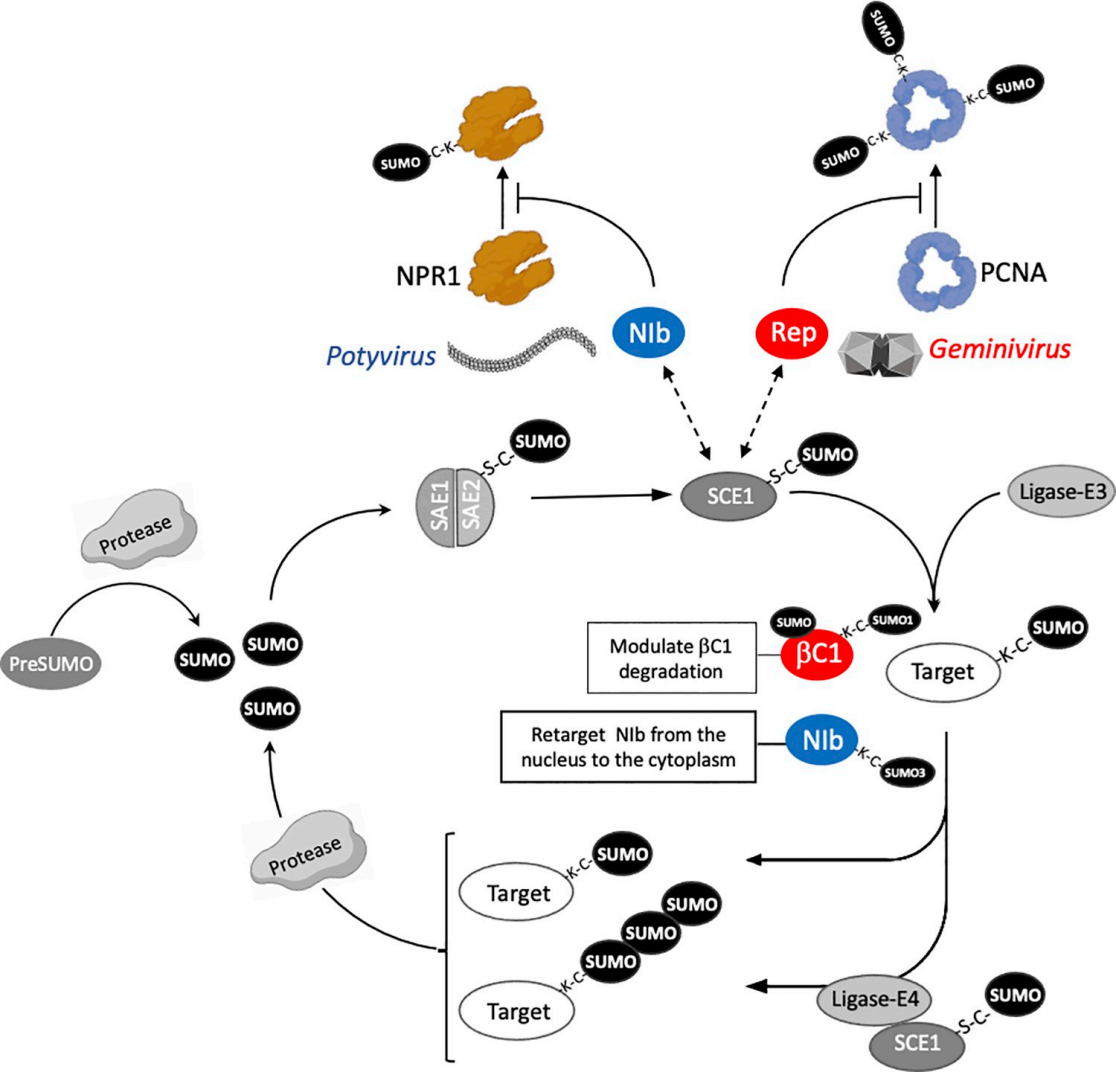
**SUMOylation pathway is targeted by proteins from plants viruses**. Synthesised SUMOs undergo C-terminal processing by SUMO proteases. Then, the mature SUMO is activated by the heterodimeric activating enzyme (SAE1/SAE2), subsequently transferred to the SUMO Conjugating Enzyme (SCE1) and covalently attached to a lysine residue of a target protein in a process that can be or not assisted by SUMO ligases (E3). Additional rounds of sumoylation by a SUMO E4 ligase enable SUMO polymer formation. SUMOylation can be reversed by a collection of SUMO proteases that cleave the linkage between SUMO and the target. Collectively, there are more proteases than SUMO ligases in plants, suggesting that the removal of this PTM is more selective than its addition. SUMO enzymes and most of the identified SUMOylation targets exhibit nuclear localization, indicating that the predominant function of this modification takes place in this cellular compartment. Proteins from geminivirus (Rep) and potyvirus (NIb) can interact with the SCE1, altering the SUMOylation of host proteins (PCNA and NPR1, respectively) or with SUMO (βC1). Degradation of the geminiviral βC1 protein is modulated by its covalent and non-covalent interaction with SUMO1. SUMOylation of the potyviral Nbl protein by SUMO3 retargeted it from the nucleus to the cytoplasm.

The SUMOylation process involves the covalent attachment of SUMO to target proteins using a hierarchical multi-enzymatic cascade similar to ubiquitination (Fig 1) [1]. This reversible modification can lead to conformational changes, alter protein interactions and influence the overall function of the modified protein, including stability, subcellular localization, and transcriptional regulation. Besides its conjugation to a target protein, SUMO is also able to non-covalently interact with many proteins harbouring SUMO interacting motifs (SIMs). Combining SUMOylation sites with SIMs, in the same or different proteins, contributes to the formation of protein macrostructures which may enhance SUMOylation by recruiting additional SUMO targets to a SUMOylation favourable environment [1]. *Arabidopsis* genome contains 8 SUMO genes, although only 4 are expressed (AtSUMO1/2/3/5). The almost identical AtSUMO1/2 are the SUMO archetype, as they are the nearest homologues of mammalian SUMO2/3. The SUMO proteins diverge in their spatial-temporal expression and functions during development and defence [2]. Plants typically express high levels of a strongly conserved SUMO isoform (AtSUMO1/2) and at least 1 weakly expressed, non-conserved isoform (AtSUMO3/5).

## Is SUMOylation involved in defence responses in plants?

SUMO is a key component of the plant immune system [3]. The changes in SUMOylation produced by the lack of SUMO E3 ligase SIZ1 (SAP AND MIZ1 DOMAIN-CONTAINING LIGASE1) or depletion of SUMO1/2 expression provoke a reduction in the susceptibility to bacteria that correlate with a higher level of salicylic acid (SA) and induction of the expression of pathogenesis-related (PR) proteins. A similar phenotype is observed in plants displaying an increase in SUMO-conjugates produced by overexpression of SUMO 1/2/3 or by the lack of function of SUMO proteases. On the contrary, SUMO3 depletion results in an increase in susceptibility to bacteria and a decrease in SA levels [4]. These results present a complex scenario in which SUMO1/2 and SUMO3 have respectively a negative and a positive role in plant immunity and where the reduction or the accumulation of SUMO conjugates cause the production of SA and the induction of systemic acquired resistance (SAR). The relevance of SUMOylation in plant defence mechanisms is additionally supported by the identification of pathogen effectors that target the SUMOylation machinery and the identification of numerous SUMO-conjugates with a role in defence against pathogens, including PAMS receptor as FLS2 (FLAGELLIN-SENSITIVE 2), NLR intracellular receptor as SCN1 (SUPERCENTIPEDE1), and positive regulators as NPR1 (NONEXPRESSOR OF PATHOGENESIS-RELATED GENES 1) [3].

## Is SUMOylation relevant for plant virus infection?

As cell intruders, viruses have developed mechanisms to modify the host SUMOylation system. Our knowledge of how viruses exploit the SUMO machinery is mainly based on studies conducted in mammalian cells [5]. Broadly, viral proteins may undergo SUMOylation or interact either with elements of the SUMO pathway or with host SUMOylated proteins. Their impact in remodelling the plant SUMOylome can vary significantly depending on the specific viral factors involved. While it was initially suggested that SUMOylation primarily played a role in the infection of nuclear-replicating viruses, recent studies have shown that the SUMO pathway is also relevant for those that replicate in the cytoplasm.

In plants, the first report of a viral protein binding to an element of the SUMO pathway, the E2 (SCE1, SUMO CONJUGATING ENZYME 1), was described for a protein, Rep, encoded by geminiviruses [6]. Geminiviruses are ssDNA viruses that replicate in the nucleus and since they do not encode a DNA polymerase, they entirely rely on the host machinery for its replication. Rep is required to initiate and terminate viral DNA synthesis and recruits the host replication machinery [7]. Mutations that impair the Rep-SCE1 interaction reduce viral DNA accumulation in planta and virus infectivity [8]. A similar negative effect was observed in plants with altered levels of SUMO (reduced by silencing or overexpression) [6]. Rep does not seem to be modified by SUMO despite its interaction with SCE1 and the presence of putative SUMOylation sites. Instead, its expression alters the SUMO conjugation of a selected subset of host proteins [8,9]. Among those, Rep reduces the SUMOylation state of plant PCNA (PROLIFERATING CELL NUCLEAR ANTIGEN), a Rep interactor and an essential cofactor involved in DNA replication, repair, and recombination [10]. The switching between these PCNA functions is modulated by PTMs, mainly ubiquitination or SUMOylation, which facilitates or hinders the interaction of PCNA with specific partners. SUMOylation of PCNA suppresses undesired recombination events between synthesised DNA molecules. In yeast, PCNA SUMOylation enrolls a DNA helicase, SRS2 (SUPPRESSOR OF RAD6 MUTANT 2), that hampers recombination by removing RAD51 (RADIATION SENSITIVE PROTEIN 51) from ssDNA and disassembling the recombination intermediate structure [11]. Mutations of PCNA that impair its SUMOylation or deletion of *Srs2* provoke an increase in homologous recombination (HR) [10]. *Arabidopsis* SRS2 interacts with plant PCNA and showed similar helicase activity to the yeast protein [12,13]. The recombination among geminiviral genomes has been widely reported and seems to result from an enhancement of the recombination frequency [14]. The relevance of HR in geminiviral infection is exposed by the fact that RAD51D promotes viral replication at the early stages of infection, and its presence is required for geminiviral recombination [15]. Those results have led to propose that the modulation of the modification status of PCNA, produced by its interaction with Rep, switches its cellular function to create an environment suitable for viral replication. The reduction of PCNA SUMOylation impairs SRS2 binding to PCNA, allowing conservation of the Rad51-ssDNA nucleoprotein filaments, that in turn will escalate the level of HR and also geminiviral replication efficiency, since HR provides a mechanism for tolerating stalled DNA replication forks [9]. Additionally, it is worth mentioning that, as in other eukaryotic PCNAs, lysine 164 of plant PCNAs is also ubiquitinated and that this PTM, unlike its SUMOylation, is not affected by the presence of Rep [12].

SUMOylation and non-covalent interaction with SUMO1 have been reported to be essential for the function of βC1 [16], a geminiviral protein, encoded by an associated beta-satellite molecule, that plays a role in symptom induction and suppression of plant defence. Considering that geminiviral βC1 is decayed either by autophagy or by the ubiquitin-proteasome system, it has been proposed that βC1 is SUMOylated as a strategy against its ubiquitin-mediated degradation. However, in accordance with the dual role of SUMO, the βC1 interaction with the SUMOylation machinery exemplifies a delicate equilibrium between defence and counter defence, in which the interaction with SUMO1 through its SIM motif promotes its degradation, while βC1 SUMOylation prevents it. Another example of the interaction between SCE1 and a plant viral factor has been described for the RNA-dependent-RNA-polymerase (NIb) encoded by a member of the single-stranded RNA virus *Potiviridae* family. Besides its interaction with the E2 enzyme, Nlb is SUMOylated by SUMO3 [17]. SUMO modification promotes viral infection by relocating NIb from the nucleus to the cytoplasm, where viral replication occurs. In accordance, mutation of the SUMOylated residue of NIb compromised virus infectivity [18]. Nevertheless, as described for the role of SUMO in the response to bacteria, this scenario is more complex since any impact in SUMO homeostasis produced by altering the levels of SUMO3 has a negative effect on viral infection. Potyviruses induce SUMO3 expression during infection, enhancing NIb SUMOylation and favouring viral replication. However, this increase in SUMO3 could also potentially trigger adverse effects on the viral infection since NPR1 SUMOylation promotes the expression of PR genes. Interestingly, the expression of NIb, but not its SUMOylated-defective version, can suppress PR expression after SUMO3 induction, indicating that NIb-SUMO suppresses the SUMO3-dependent activation of NPR1 [18]. The mechanism behind this effect has been recently shown since NIb also binds NPR1 and prevents its SUMO modification [19]. This interaction also impedes the phosphorylation of NPR1, an additional PTM essential for NPR1 to strongly reprogram the transcription during immune responses.

Finally, factors involved in general RNA decay have been probed to play a role in the plant viral defence [20]. Recently, Ge and collaborators showed that turnip mosaic virus (TuMV) RNA is degraded by the action of PELOTA and HSB1, 2 factors involved in non-stop (NSD) and no-go xRNA decay (NGD) [21]. Viral RNA decay depends on PELOTA recognition of a motif in the TuMV genome required to induce transcriptional slippage and its interaction with HSB1. The authors revealed that the SUMOylation of PELOTA is required for binding HSB, giving evidence for the first time of the role that SUMO plays on the performance of the RNA decay that can participate in the degradation of the viral RNAs in plants.

## Concluding remarks

In sum, the SUMOylation pathway in plants plays a complex and dual role in the context of viral infections, contributing to the defence against viruses and being exploited by viruses to promote their own replication and evasion strategies. Understanding these interactions at a deeper level can lead to significant advancements in plant virology and agricultural biotechnology.
